# Antarctic Soil Yeasts with Fermentative Capacity and Potential for the Wine Industry

**DOI:** 10.3390/foods12244496

**Published:** 2023-12-16

**Authors:** Laura Navarro, Mariona Gil i Cortiella, Ana Gutiérrez-Moraga, Nancy Calisto, Cristina Ubeda, Gino Corsini

**Affiliations:** 1 Instituto de Ciencias Biomédicas, Facultad de Ciencias de la Salud, Universidad Autónoma de Chile, Santiago 8900000, Chile; laura.navarro@uautonoma.cl (L.N.); ana.gutierrez@uautonoma.cl (A.G.-M.); nancy.calisto@umag.cl (N.C.); 2 Instituto de Ciencias Aplicadas, Facultad de Ingeniería, Universidad Autónoma de Chile, Santiago 8900000, Chile; mariona.gil@uautonoma.cl; 3 Departamento de Producción Agropecuaria, Facultad de Ciencias Agropecuarias y Medioambiente, Universidad de La Frontera, Temuco 4811230, Chile; 4 Centro de Investigación y Monitoreo Ambiental Antártico (CIMAA), Departamento de Ingeniería Química, Universidad de Magallanes, Avenida Bulnes 01855, Punta Arenas 6210427, Chile; 5 Departamento de Nutrición, Bromatología, Toxicología y Medicina Legal, Facultad de Farmacia, Universidad de Sevilla, C/P. García González No 2, 41012 Sevilla, Spain

**Keywords:** Antarctic yeast, fermentative yeast, psychrophilic yeast, wine, *Mrakia*

## Abstract

Low fermentation temperatures are usually employed to obtain high-quality wines. This is especially interesting for white wine production since it prevents the loss of volatile compounds and a browning appearance; however, available fermentative yeasts do not usually tolerate low temperatures. Therefore, an interesting place to find new yeasts with cryotolerance is the Antarctic continent. From soil samples collected in Antarctica, 125 yeasts were isolated, of which 25 exhibited fermentative activity at 10 °C. After a fingerprinting assay, we classified the candidates into nine isotypes and sequenced internal transcribed spacer regions for their identification. These yeasts were identified as part of the *Mrakia* genus. Sugar and alcohol tolerance tests showed that some of these Antarctic soil yeasts were able to grow up to 9% alcohol, and 25% sugar was reached; however, they exhibited longer latency periods compared to the control *Saccharomyces cerevisiae*. The optimal growing temperature for the isolated Antarctic yeasts was between 10 °C and 15 °C. A comprehensive analysis of the results obtained showed that the isolates 10M3-1, 4M3-6, and 4B1-35 could be good candidates for fermentation purposes due to their alcohol, sugar tolerance, and growth features. Our results prove that it is possible to isolate fermentative yeasts from Antarctic soil with promising characteristics for their potential use in the wine production industry.

## 1. Introduction

Wine has been produced since ancient times, and today it continues to expand to many different places all over the world. Due to this, the wine industry has become a highly competitive market, with several wineries increasing their experimentation to produce wines with distinguishable features to diversify and create new products. Multiple factors modulate the characteristics of a wine, such as weather, soil, vineyard practices, winemaking practices/conditions, microorganisms involved in the process, etc. [1,2,3]. Among these factors, the yeast employed to carry out the fermentation is one of the elements that has a significant impact on the final product, especially in terms of the aromatic characteristics of the wine [4]. Therefore, wineries are currently focusing on the search for novel fermentative yeast strains or the utilization of non-traditional options to obtain wines with greater typicality [5]. In the case of the food industry, the most widely used yeasts are of the Saccharomyces type. They are employed to carry out fermentation processes, for example, to produce alcoholic beverages such as wine and beer. These yeasts can endure the specific conditions of this process, i.e., high osmolarity, high ethanol content, and low pH [6,7]. On the other hand, non-*Saccharomyces* yeast strains are less used in alcoholic beverage production, mainly due to their challenges in surviving high concentrations of alcohol produced during fermentation [8]. However, the interest in this kind of yeast in the industry has grown, as it has been observed that they have a positive effect on the organoleptic characteristics, increasing aromatic complexity and enhancing wine quality [9,10,11] when used along with *Saccharomyces* strains in co-inoculation experiments. Some authors have successfully tested some non-Saccharomyces yeast strains from the gender *Metschnikowia, Kluyveromyces*, or *Candida* to produce wines with lower alcohol levels [3,12,13]. In addition to the yeast strain employed for alcoholic fermentation, there are other critical conditions during wine production that directly influence the organoleptic properties of this beverage. The temperature reached during the alcoholic fermentation strongly affects the organoleptic characteristics of the final product, especially in the case of white wines [6,14,15,16,17]. This is partially due to low temperatures producing wines with a higher aromatic potential due to a lower loss of aromas by evaporation. It has been observed that lower temperatures during fermentation affect white, rosé, and red wines in a similar way, resulting in wines with more fruity and floral profiles. This effect is primarily due to the increased production of esters, making the wines more aromatic and complex [14,16,18,19]. Moreover, conducting alcoholic fermentation at lower temperatures also prevents the appearance of undesirable browning. This oxidative process involves sugars, lipids, amino acids, and phenols [20,21], leading to a decrease in the sensory quality of wines (loss of color, flavor, and aroma) and an increase in astringency [22]. Despite the advantages of a low fermentation temperature, conducting alcoholic fermentation under these conditions also entails some drawbacks. Yeast may stop growing or die due to the low temperatures, ceasing to be the dominant culture, which creates an opportunity for other microorganisms, such as fungi or bacteria, to take advantage and colonize the environment, leading to wine spoilage.

Metabolic enzymes present in yeasts work in a concrete range of temperatures. The typically employed yeast in the wine industry, *Saccharomyces cerevisiae*, has an optimal growth temperature of around 32 °C; therefore, the fermentation temperature is always above 15 °C [23]. As a result, the fermentation temperature during wine production is a compromise between the quality of the wine and the viability of the microorganism. Accordingly, the selection of yeast strains with the ability to ferment grape must, at low temperatures, leads to less-oxidized wines with a high concentration of volatile compounds. Hence, the isolation of a new fermentative yeast from low-temperature climates could be a proper approach [24]. The Antarctic media have a wide microbial diversity, enabling the isolation of adequate candidates. Most microorganisms studied in cold environments are bacteria [25,26,27], but it has been suggested that yeasts could be better adapted to low temperatures than bacteria [28]. Yeast has been isolated from the Arctic, Alpine glaciers, Antarctic ecosystems, and Argentinean Patagonia [17,24,29,30,31,32,33]. Various cold adaptation features have been described in Antarctic yeast. These include a shift from a respiratory metabolism at 23 °C to a fermentative metabolism when grown in cold conditions (0 °C), achieved through the downregulation of citrate cycle genes and the ETC genes of *Rhodotorula frigidialcoholis* [34]. The metabolic enzymes of the yeast strains isolated from Antarctic media are expected to work at low temperatures. Some of these cold-active enzymes are well-known, being an attractive research topic in many fields [35]. They tend to share similar characteristics, such as improved flexibility and thermal compensation of the yeast [36].

In the search for fermentative yeast, Ballester-Tomás et al. (2017) [37] implemented a study of a strain of *Candida sake* isolated from the sub-Antarctic region. They compared some volatile compounds in the wines fermented with *Saccharomyces cerevisiae* strain QA23 to the compounds in wines produced with *C. sake*. They observed that wines fermented with *C. sake* had a lower ethanol and glycerol content, whereas the wines fermented with this sub-Antarctic strain presented larger amounts of higher alcohols.

While it is well-known that fermenting grape juice at low temperatures significantly enhances the final quality of white wine, and extensive research has been conducted on Antarctic yeasts, little is known about the potential of psychrophilic Antarctic yeasts as fermenting agents. Therefore, the objectives of the present study were to isolate and identify yeasts from different Antarctic locations, for potential use in the wine industry, with the ability to ferment at various temperatures and resistance against different alcohol concentrations and osmotic pressures.

## 2. Materials and Methods

### 2.1. Yeast Isolation

Six soil samples collected from Fildes Bay (62°11′ S, 58°57′ W) and three soil samples from Collins Glacier (62°9743′ S; 58°27,978′ W) were processed for yeast isolation. The soil was suspended in sterile water, and inoculums were seeded into YM agar plates (3% yeast extract (Becton Dickinson and Company, Pont de Claix, France), 3% malt extract (Becton Dickinson and Company, Pont de Claix, France), 5% peptone (Merck, Darmstadts, Germany), 2% agar (Becton Dickinson and Company, Pont de Claix, France), supplemented with 1% of glucose (Sigma-Aldrich Co., St. Louis, MO, USA) and 100 μg/mL of kanamycin (Caisson Labs, Smithfield, UT, USA) and ampicillin. Plates were cultured at 4, 10, and 18 °C. Non-filamentous colonies were selected and transferred into fresh YM agar plates (1% glucose). Isolates were analyzed macroscopically and microscopically for subsequent confirmation of their yeast nature.

### 2.2. Macroscopic and Microscopic Analyses

The colony growth in YM agar plates with 1% glucose (Sigma-Aldrich Co., St. Louis, MO, USA) of different microorganisms isolated was analyzed and registered using an optical microscope Motic BA310 with a 40× objective and an additional 4.5× magnification for imaging capture [38].

### 2.3. Fermentative Capacity Test

To determine the fermentative capacity, we used a modified YM medium containing 3% yeast extract (Becton Dickinson and Company, Pont de Claix, France), 3% malt extract (Becton Dickinson and Company, Pont de Claix, France), 5% peptone (Merck, Darmstadts, Germany), and 2% agar (Becton Dickinson and Company, Pont de Claix, France). The medium was supplemented with lactose (Sigma-Aldrich Co., St. Louis, MO, USA) and sucrose (Sigma-Aldrich Co., St. Louis, MO, USA) at a concentration of 10 g/L, 1 g/L of glucose, and 25 mg/L of phenol red—a pH variation indicator (Sigma-Aldrich Co., St. Louis, MO, USA). In assay tubes, the media were inoculated with the respective Antarctic yeast strains, and these were cultured at 10 °C for 14 days. As a control yeast for the fermentative test, *Saccharomyces cerevisiae* EC-1118 (Lallemand-South America, Montreal, QC, Canada) was employed.

### 2.4. Sugar and Alcohol Tolerance Test

The sugar tolerance capacity of the yeasts was analyzed with a multi-plate culture test with the YM medium, supplemented with different concentrations of glucose and fructose (Sigma-Aldrich Co., St. Louis, MO, USA) in a 1:1 ratio. These sugar concentration ranges started with 5% *w*/*v*, increasing by 5% until reaching a monosaccharide concentration of 25% w/v. Additionally, tolerance to alcohol was determined using cultures with ethanol (Sigma-Aldrich Co., St. Louis, MO, USA) at 0, 3, 6, and 9% *v*/*v*. Tolerance was determined by measuring the optical density of the samples at 600 nm every 48 h for 8 days. As a control yeast for the sugar and tolerance tests, *Saccharomyces cerevisiae* EC-1118 (Lallemand-South America) was employed.

### 2.5. DNA Isolation

To isolate the genomic DNA, the following protocol was performed. The samples were obtained from a culture plate and solved in 200 µL of sterile water. Then, 400 µL of lyticase enzyme (Sigma-Aldrich Co., St. Louis, MO, USA) at a concentration of 1000 U/mL was added. The samples were incubated at 30 °C for 2 h. Then, 10 µL of proteinase K (Promega, Madison, WI, USA) at a concentration of 20 mg/mL was added and the samples were incubated for 2 h at 50 °C. A heat shock protocol was performed twice: 30 min at −20 °C and then 10 min at 80 °C. The samples were then vortexed at the maximum speed for 5 min. Next, the Zymo Research Quick-DNA Fungal and Bacterial miniprep kit Cat.No.: D6005 (Zymo Reasearch, Irvine, CA, USA) was used following the instructions of the manufacturer.

### 2.6. Molecular Characterization of Antarctic Fermentative Yeasts

The arbitrary primers used for fingerprinting corresponded to (GTG)5 and (GACA)4. The reaction mix for the PCR contained 50 µL: 25 µL GoTaq Green Master Mix from Promega, provided by Fermelo Biotech (Región Metropolitana, Chile); 1 uL of each primer at 1 nM; 2 µL of genomic DNA; and water to reach a total reaction volume of 50 µL. The PCR reaction started with an initial denaturation cycle at 95 °C for 3 min, followed by 45 cycles at 95 °C for 1 min for denaturation, 50 °C for 1 min for annealing, and 72 °C for 90 s for extension, with a final extension at 72 °C for 10 min.

For the identification of the samples, the amplification and sequencing of the internal transcribed spacer (ITS) were carried out. The primers used in the amplification of the ITS region were ITS1 (5′-TCCGTAGGTGAACCTGCGG-3′) and ITS4 (5′-TCCTCCGCTTATTGATATGC-3). To amplify the ITS region, the reaction mix for the PCR contained 50 µL: 20 µL GoTaq Green Master Mix from Promega, provided by Fermelo Biotech (Chile); 1 µL of each primer at 1 nM; 2 µL of DNA; and 19 µL of water to reach a total reaction volume of 50 µL. The PCR reaction started with an initial denaturation cycle at 94 °C for 5 min, followed by 45 cycles at 94 °C for 1 min, 50 °C for 1 min for annealing, and an extension at 72 °C for 2 min, with a final extension at 72 °C for 5 min. The PCR products were purified for sequencing purposes using the E.Z.N.A. Gel Extraction Kit from Omega Biotech, provided by Grupo SIBI (Santiago, Chile). The results from the amplification of the ITS region were analyzed using the Basic Local Alignment Search Tool (BLAST), optimized for highly similar sequences.

### 2.7. Optimal Growth Temperature

The growth of each selected yeast was analyzed in both solid and liquid media. In the liquid media, growth was determined using optical density. An initial culture was used to inoculate the samples of the YM medium with 100 µL in a total volume of 8 mL; each culture was performed in triplicate. The cultures were then incubated at 4, 10, 15, 18, and 21 °C. The optical density was measured at 600 nm every 48 h. Growth in the solid medium was also analyzed. For this purpose, YM plates were prepared, and a 5 µL spot of each Antarctic yeast was placed in duplicate. The plates were cultured at 4, 10, 15, 18, and 21 °C. The plates were analyzed for colony growth every 48 h for 2 weeks. As a control yeast for optimal growth temperature determination, *Saccharomyces cerevisiae* EC-1118 (Lallemand-South America) was employed.

## 3. Results

### 3.1. Yeast Isolation

**Figure 1:**
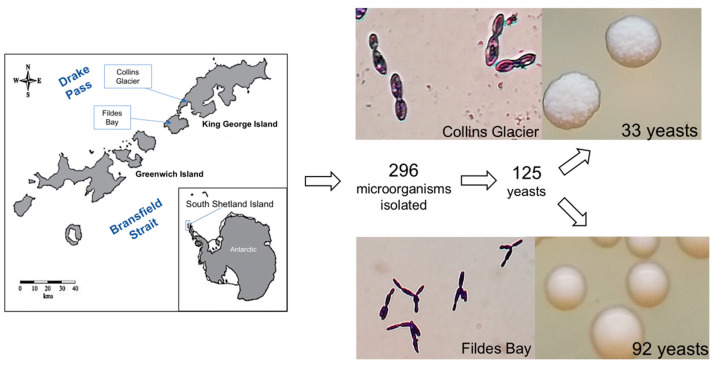
Process of isolation of the yeasts from soil sampling areas in Antarctica, Collins Glacier, and Fildes Bay, and the results of the isolation process, including images showing an example of the isolated yeasts from each location.

From all the soil samples, a total of 296 microorganisms were isolated. Following the macro and microscopic analyses, it was determined that 125 of the samples were indeed yeasts, as indicated by the observed cell size in microscopy (Figure 1). All the yeast was isolated from cultures at 4 and 10 °C; at 18 °C, no yeast was isolated.

### 3.2. Fermentative Capacity Test

**Figure 2:**
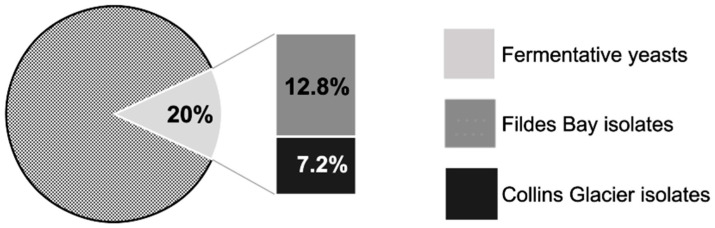
Percentage of fermentative yeasts of each soil sample from Collins Glacier and Fildes Bay.

The 125 yeasts isolated were analyzed to determine their fermentative capacity. This was performed at 10 °C with a pH variation indicator. This indicator turns from red at a neutral pH to yellow at an acidic pH. In fermentation processes, the reduction of the pH value is characteristic. From the 125 yeast samples, 25 (20%) were capable of fermenting optimally at 10 °C. Out of these 125 yeasts, 12.8% of the fermentative isolates (16 isolates) were obtained from the soil sample from Fildes Bay, and 7.2% (9 isolates) were from the Collins Glacier (Figure 2). This indicates that we recovered a greater number of fermentative yeasts in the soil of the Collins Glacier (9/33, 27.2%) compared to the soil of Fildes Bay (16/92, 17.4%).

### 3.3. Molecular Characterization of Antarctic Fermentative Yeasts

**Figure 3:**
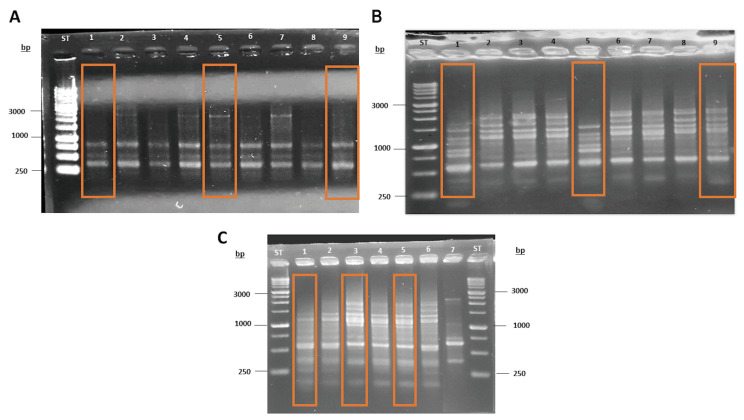
Fingerprinting assay to discard overrepresentation of the isolates. (**A**) Yeasts 10M3-1 (1), 10M3-4 (2), 10M3-9 (3), 10M3-13 (4), 10M3-19 (5), 10M3-20 (6), 10M3-21 (7), 10M3-22 (8) and 4M3-6 (9); (**B**) Yeasts 4B1-19 (1), 4B1-20 (2), 4B1-21 (3), 4B1-22 (4), 4B1-23 (5), 4B1-24 (6), 4B1-25 (7), 4B1-26 (8) and 4B1-35 (9); and (**C**) Yeasts 4C2-38 (1), 4C2-39 (2), 10C2-3 (3), 10C2-4 (4), 10B2-15 (5), 10B2-13 (6) and *S. cerevisiae* EC1118 (7) Selected yeasts are highlighted by a rectangle in each respective lane.

As a first step in the molecular characterization of the Antarctic fermentative yeasts, we conducted a fingerprinting analysis using the primers (GACA)4 and (GTG)5. This methodology was used to determine which of the 25 strains were identical and, therefore, rule them out. This allowed us to select only nine strains from the 25 isolates, using repetitive region DNA profile analyses by PCR fingerprinting (Figure 3).

**Figure 4:**
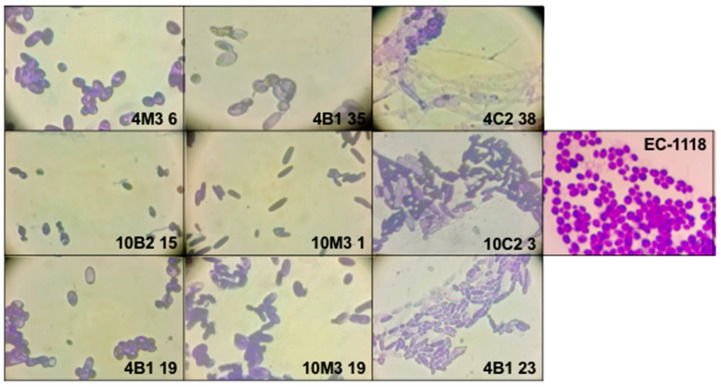
Optic microscopy of the 9 isotypes using a total amplification of 1800×. Graphical scale 10 μm.

We identified nine isotypes (Table 1), each represented by a specific strain: 4B1-35, 4B1-23, 4B1-19, 4C2-38, 10C2-3, 10B2-15, 10M3-1, 10M3-19, and 4M3-6. The results are summarized in Table 1. Also, these yeasts were analyzed by optic microscopy, using an amplification of 400× and an additional amplification of 4.5×, resulting in a total magnification of 1800× (Figure 4).

In addition, these nine representative yeast strains of different isotypes were analyzed by amplification and sequencing of the 18S rRNA ITS region using primers IT1 and ITS4 (Table 2). The results were obtained using BLAST and showed that these Antarctic yeast strains belong to the *Mrakia* genus, although some samples resulted in more than a single hit in the database with the maximum identity percentage and query cover.

### 3.4. Sugar and Alcohol Tolerance of Antarctic Fermentative Yeasts

**Figure 5:**
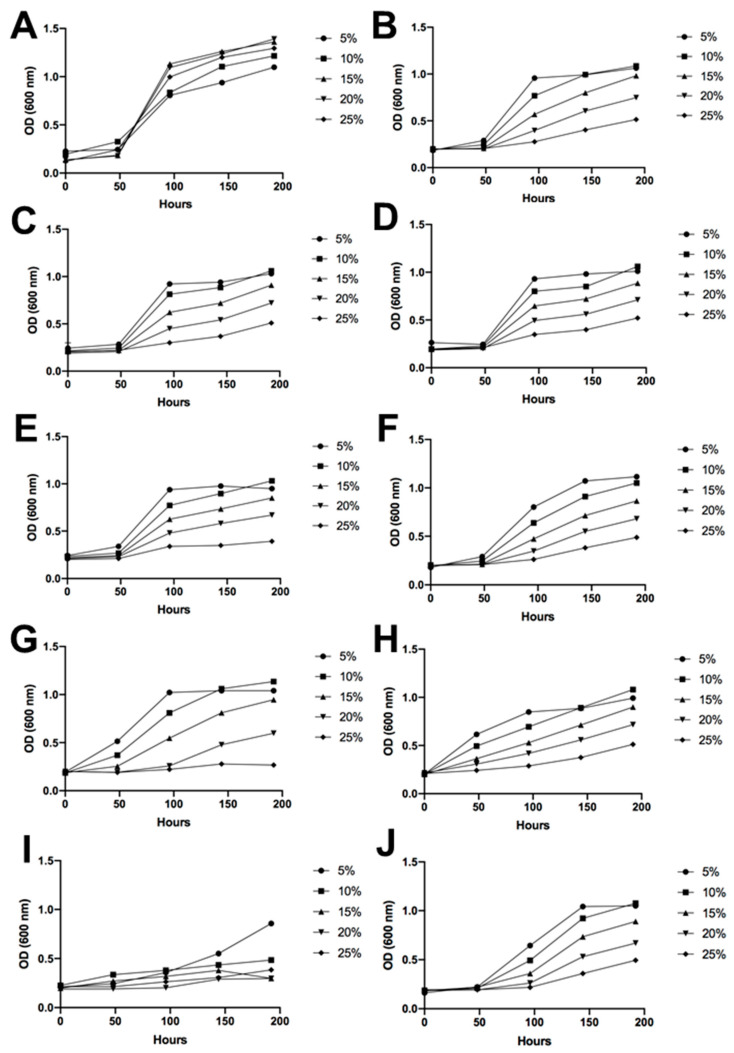
Sugar tolerance. Yeast growth represented by the optical density under a wide range of sugar concentrations in the culture medium, ranging from 5% *w*/*v* to 25% *w*/*v*. (**A**) *Saccharomyces cerevisiae* EC-1118, (**B**) I1, (**C**) I2, (**D**) I3, (**E**) I4, (**F**) I5, (**G**) I6, (**H**) I7, (**I**) I8, and (**J**) I9.

**Figure 6:**
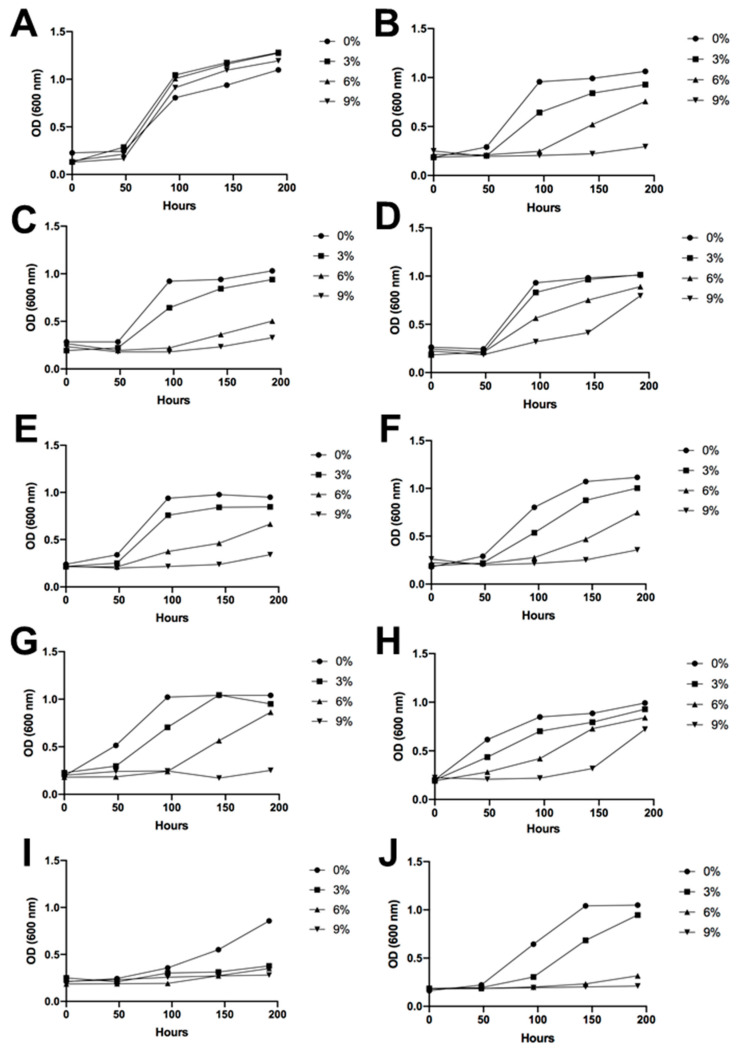
Ethanol tolerance. Yeast growth is represented by the optical density under a wide range of alcohol concentrations in the culture medium, ranging from 0% to 9% *v*/*v*. (**A**) *Saccharomyces cerevisiae* EC-1118, (**B**) I1, (**C**) I2, (**D**) I3, (**E**) I4, (**F**) I5, (**G**) I6, (**H**) I7, (**I**) I8, and (**J**) I9.

The Antarctic fermentative yeasts were subjected to an assay to assess sugar and alcohol tolerance. The nine different isolated yeasts withstood the experimental growing conditions, displaying a longer latency period and slower growth or proliferation compared to the control, *Saccharomyces cerevisiae* EC-1118. However, as the data show, there was an increase in the optical density (OD) over time in every condition. Therefore, there was observable growth, and the isolated Antarctic yeasts exhibited tolerance to conditions of high sugar content and high alcohol concentration (Figure 5 and Figure 6).

As can be observed in Figure 5, the typical yeast employed in wine production, *S. cerevisiae*, grew quickly in all tested sugar concentrations. In general, the isolated strains showed a shorter latency period at the lowest sugar concentration tested in the medium. Strains 10M3-1, 4M3-6, 4B1-35, 4C2-38, 10B2-15, and 10C-23 showed a similar growth pattern across different sugar percentages. The strain 4B1-19 exhibited a longer latency period compared to the aforementioned yeasts; however, by the end of the experiment, its optical density was similar to that of the other isolates. On the contrary, the strains 10C-23 and 10M3-19 initiated growth after 50 h, even before *S. cerevisiae*. Finally, the highest lability to sugar in the medium was observed in strain 4B1-23, which only grew at a concentration of 5% sugar (*w*/*v*) (Figure 5).

Regarding alcohol tolerance, in general, all the Antarctic yeasts presented a higher difficulty for growth in an ethanolic medium compared to the wine yeast *S. cerevisiae*. However, under an ethanol concentration of 6% *v*/*v*, most of the isolated Antarctic yeasts proved to be able to grow, exhibiting a constant increase in OD up to the final day of measurements, apart from 4B31-19 and 4B1-23 (Figure 6). These two yeasts presented the lowest resistance to ethanol in the media. At 9% ethanol, strains such as 10M3-19 and 4B1-35 showed growth at the end of the measurements, indicating an increasing similarity to the control without ethanol.

### 3.5. Optimal Growth Temperature

**Figure 7:**
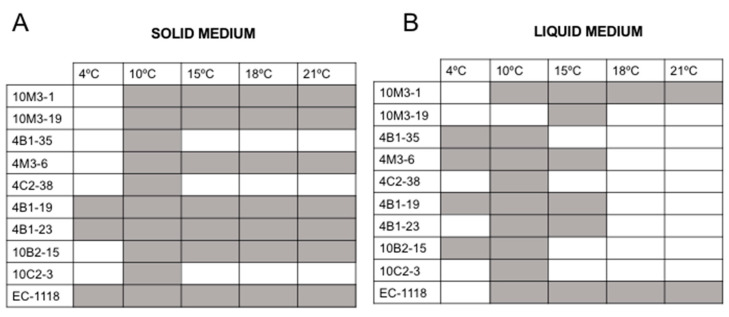
Optimal growth temperature. Testing of 5 different growth temperatures for the 9 Antarctic fermentative yeasts and EC1118 grown in solid (**A**) or liquid (**B**) medium.

To determine the optimal growth temperature of each yeast, they were grown at five different temperatures (4, 10, 15, 18, and 21 °C), with the optical density of the samples measured every 48 h. It was remarkable how, at 4 °C, most of the yeasts were unable to grow, especially considering that these were isolated from Antarctic soil samples. The growth tendency was better at 10 °C and 15 °C. The Antarctic yeast 10M3-1 showed a surprising ability to grow at high temperatures, comparable to the control yeast (Figure 7). It is important to highlight that the Antarctic isolates have a longer latency period in comparison to the commercial yeast *Saccharomyces cerevisiae* EC1118 from Lallemand.

## 4. Discussion

The nine different fermentative yeasts demonstrated their considerable potential to produce alcoholic beverages, such as wine, as they were able to grow in the presence of alcohol and high concentrations of sugar in the culture media. However, as expected, *S. cerevisiae* exhibited the best growth performance because it is perfectly adapted to these conditions. Therefore, unlike *S. cerevisiae* EC-1118, the Antarctic strains isolated have a longer latency period. However, all the samples demonstrated tolerance to high concentrations of sugar and considerable amounts of ethanol. This is noteworthy, especially considering that these are environmental yeasts derived from soil (Figure 5 and Figure 6). It is important to note that the alcohol tolerance tests were conducted with ethanol shock, meaning that the growing media already contained the alcohol content when the yeasts were placed in the plates. The progressive increase of ethanol in the media, as it naturally occurs during the fermentation process, would probably enable the Antarctic yeast to undergo an adaptation period, leading to better performance results. Despite this, some yeasts exceeded expectations and grew under a 9% ethanol shock. Of note are strains 10M3-19 and 4B1-35, which showed growth at the end of the measurements, thus being strong candidates for grape must fermentation. Given that all the identified Antarctic isolated yeasts were non-*Saccharomyces*, these results were expected since, in comparison with *Saccharomyces* yeasts, their alcohol resistance is usually lower [39]. A study focused on *Candida sake* isolated from Antarctica demonstrated growth with 3% ethanol in the media, but only *S. cerevisiae* had the ability to grow with 5 or 9% alcohol [37]. Moreover, considering that non-*Saccharomyces* yeasts are usually employed at the beginning of sequential inoculations [40], some of the isolated yeasts of this study would be suitable candidates for this fermentation strategy.

The resistance of the Antarctic yeasts isolated to the osmotic pressure of high sugar concentrations was not as satisfactory as their alcohol tolerance, as all of them exhibited a very long latency period. However, considering a grape must with a 20% sugar content, all of them, apart from 4C2-38 and 4B1-23, could be useful, albeit with the drawback of a longer fermentation. The resistance of some of the isolated yeasts to high sugar and alcohol concentrations demonstrates the adaptation ability that these fermentative yeasts possess, likely acquired from growing in an extreme environment such as Antarctica [41].

The ITS region sequencing demonstrated that the isolates were non-*Saccharomyces*, and all the fermentative Antarctic yeasts belong to the *Mrakia* genus (Table 2). However, some samples resulted in more than one hit with 100% identity and query cover, as was the case for isolates 4B1-35, 4C2-38, 10C2-3, 10M3-1, 10M3-19, and 4M3-6, which showed identities as both *M. blollopsis* and *M. psychrophilia*. For isolates 4B1-23, 4B1-19, and 10B2-15, the results suggest a possible relation to the *M. robertii* species. It is relevant to highlight that more molecular analyses should be performed in the future. The database used for this determination has some limitations due to the incredible variability in fungus genomes. Therefore, the incorporation of analysis regarding other conserved genetic regions in the ribosomal DNA can be performed to bolster these results [42], including entire genome sequencing.

In the literature, there are precedents that show the use of environmental species for alcoholic beverage production. Among the *Mrakia* genus, there are several species that can ferment glucose and produce ethanol [43,44]. Tsuji et al. (2018) [45] described several species from this genus isolated from Arctic environments. Very recently, Linnakoski et al. (2023) [46] studied *Mrakia gelida* isolated from Finnish forest environments with brewing purposes. In fact, the psychrophilic strain of *M. gelida* has been used to produce beer on a pilot scale, and the authors stated that the *M. gelida* beer showed a better sensory profile than that of *Saccharomycodes ludwigii* [47].

The analysis of the optimal growth temperature showed that only a few Antarctic yeasts grew at 4 °C. Nevertheless, the isolates grew better at 10 °C and 15 °C. This result aligns with the findings by Ballester-Thomas et al. (2017) [37] in *Candida sake* isolated from Antarctica, which exhibited optimal growth at 12 °C. Among the Antarctic yeast tested, 10M3-1 and 10B2-15 grew at 21 °C, particularly in solid media (Figure 7). However, it is possible that in a liquid medium, the latency period could be higher, which might be consistent with the source of origin of the samples. Therefore, an acclimation process could help reduce this detrimental characteristic. Antarctica has a strongly seasonal climate characterized by continuous freezes and thaws. These changes are reflected in the population, density, and distribution of microorganisms [48]. Therefore, it is not unrealistic that these microorganisms have been selected by environmental pressure and are able to grow at a different range of temperatures, especially the samples from the Collins Glacier, which were obtained from the rhizosphere of *Deschampsia antarctica* Desv., one of the two vascular plants that grow in Antarctica. Clearly, the microclimate existing in the rhizosphere differs from the common soil. The samples from Fildes Bay were obtained at a 4 or 6 cm depth and resulted in the isolation of the largest percentage of fermentative yeasts (66.7%).

As mentioned above, these are not the first yeasts isolated from the Antarctic for oenological purposes. In the work of Ballester-Thomas et al. (2017) [37], a cold-tolerant *Candida sake* H14Cs was isolated for grape must fermentation. The use of this yeast for fermentation gave rise to a fermented must with low ethanol and glycerol contents and different aromatic features compared to the must fermented with QA23 *S. cerevisiae* yeast. Nonetheless, *Candida sake* H14Cs showed high sensitivity to ethanol toxicity, making it difficult to use this yeast in winemaking. However, this disadvantage can be addressed by considering the use of mixed cultures with traditional wine yeast strains, such as *S. cerevisiae* [37]. The latter consideration applies to our findings as well. Given the long latency period and alcohol tolerance of the isolated yeasts from the Antarctic soil samples, it might be useful to use mixed cultures to produce a more distinctive wine with these Antarctic yeasts.

In summary, considering the ability of some of the yeasts isolated in this study to grow at low temperatures, thrive in low sugar content in the media, and exhibit slow but constant growth when exposed to an ethanol shock of 9%, they emerge as very interesting candidates for the production of certain types of wines. In this sense, sparkling wine production endorses these conditions during the second fermentation [49]; therefore, these yeasts could be potential candidates to carry out this production process.

## 5. Conclusions

The methodology employed allowed the isolation of yeast from soil samples collected from King George Island and Fildes Bay in Antarctica. Out of the 125 yeasts isolated, 25 of them demonstrated fermentative capacity. After the PCR fingerprinting, nine of them showed to be different isotypes, and some were successfully identified. Isotypes 2, 3, and 6 were identified with *M. robertii* species, while isotypes 1, 4, 5, 7, 8, and 9 could correspond to *M. blollopsis* or *M. psychrophilia* species. Moreover, the fermentative yeast showed characteristics such as high alcohol and sugar tolerance, which positioned them as possible candidates to be fermentative yeasts for the wine industry. Furthermore, their ability to grow at a wide temperature range proved to be interesting, with optimal growth temperatures between 10 and 15 °C. A few of them even demonstrated the ability to grow at 4 °C in vitro.

Antarctica, like any other extreme environment, has a huge potential for the discovery of different microorganisms with diverse properties. Future research will focus on must fermentation employing these yeasts at low temperatures to deeply analyze their potential as yeast for wine production.

## Figures and Tables

**Table 1:** Classification of isolated yeasts using the ITS region sequencing.

Isotype	Yeasts	Selected Yeast
I1	4B1-26, 4B1-25, 4B1-24, 4B1-22, 4B1-21, and 4B1-20	4B1-35
I2	4B1-23	4B1-23
I3	4B1-19	4B1-19
I4	4C2-38, 4C2-39, and 4C2-4	4C2-38
I5	10B2-13 and 10C2-3	10C2-3
I6	10B2-15	10B2-15
I7	10M3-1, 10M3-4, 10M3-9, 10M3-12, 10M3-21, and 10M3-22	10M3-1
I8	10M3-19	10M3-19
I9	4M3-6 and 10M3-20	4M3-6

**Table 2:** Identification of the Antarctic isolates using bioinformatics.

Yeast (Isotype)	Identification	Isotype ITS Length (bp)/GenBank Sequence Length (bp)	Identity (%)/Covered (%)	GenBank Accession Number
4B1-35 (I1)	Mrakia blollopis CPCC 300333	550/639	100/100	MZ683240.1
	Mrakia psychrophilia T5Mp	550/595	100/100	JQ857018.1
	Mrakia psychrophilia AS 2.1971	550/601	100/100	EU224267.1
	Mrakia psychrophilia AB16-13	550/610	99.81/100	LC125322.1
	Mrakia psychrophilia BF-OTU180	550/813	99.81/100	AM901861.1
4B1-23 (I2)	Mrakia robertii YH18	504/ 635	100/100	MT048630.1
	Mrakia robertii YH01	504/631	100/100	MT048621.1
	Mrakia robertii GR2-2-4-9	504/1207	100/100	LC514985.1
	Mrakia robertii A2(b)	504/ 1210	100/100	MH481688.1
	Mrakia robertii AI	504/1212	100/100	MH481684.1
4B1-19 (I3)	Mrakia robertii YH18	513/ 635	100/100	MT048630.1
	Mrakia robertii YH01	513/631	100/100	MT048621.1
	Mrakia robertii GR2-2-4-9	513/1207	100/100	LC514985.1
	Mrakia robertii A2(b)	513/1210	100/100	MH481688.1
	Mrakia robertii AI	513/1212	100/100	MH481684.1
4C2-38 (I4)	Mrakia blollopis CPCC 300333	491/639	100/100	MZ683240.1
	Mrakia sp. I12F-02259	491/619	100/100	JX852329.1
	Mrakia psychrophilia T5Mp	491/595	100/100	JQ857018.1
	Mrakia psychrophilia AS 2.1971	491/601	100/100	EU224267.1
	Mrakia psychrophilia AB16-13	491/610	99.80/100	LC125322.1
10C2-3 (I5)	Mrakia blollopis CPCC 300333	513/639	100/100	MZ683240.1
	Mrakia psychrophilia AB16-13	513/610	99.81/100	LC125322.1
	Mrakia blollopis MKOTU31	513/606	99.60/100	KP714624.1
	Mrakia psychrophilia AB16-12	513/606	99.61/100	LC125311.1
	Mrakia psychrophilia AS 2.1971	513/601	100/98	EU224267.1
10B2-15 (I6)	Mrakia robertii YH18	554/ 635	100/100	MT048630.1
	Mrakia robertii YH01	554/631	100/100	MT048621.1
	Mrakia robertii GR2-2-4-9	554/1207	100/100	LC514985.1
	Mrakia robertii A2(b)	554/ 1210	100/100	MH481688.1
	Mrakia sp. isolate J-39	554/1212	100/100	KY782313.1
10M3-1 (I7)	Mrakia blollopis CPCC 300333	528/639	100/100	MZ683240.1
	Mrakia psychrophilia BF-OTU180	528/813	99.82/100	AM901861.1
	Mrakia psychrophilia AB16-13	528/610	99.82/100	LC125322.1
	Mrakia psychrophilia AB16-2	528/610	99.64/100	LC125311.1
	Mrakia psychrophilia T5Mp	528/595	100/98	JQ857018.1
10M3-19 (I8)	Mrakia blollopis CPCC 300333	502/639	100/100	MZ683240.1
	Mrakia psychrophilia T5Mp	502/595	100/100	JQ857018.1
	Mrakia psychrophilia AS 2.1971	502/601	100/100	EU224267.1
	Mrakia psychrophilia AB16-13	502/610	99.80/100	LC125322.1
	Mrakia psychrophilia BF-OTU180	502/813	99.80/100	AM901861.1
4M3-6 (I9)	Mrakia blollopis CPCC 300333	492/639	100/100	MZ683240.1
	Mrakia sp. I12F-02259	492/619	100/100	JX852329.1
	Mrakia psychrophilia T5Mp	492/595	100/100	JQ857018.1
	Mrakia psychrophilia AS 2.1971	492/601	100/100	EU224267.1
	Mrakia blollopis MKOTU31	492/606	99.50/100	KP714624.1

## Data Availability

Data is contained within the article.

## References

[1] Van Leeuwen C., Seguin G. (2006). The concept of terroir in viticulture. J. Wine Res..

[2] Hernandez-Orte P., Concejero B., Astrain J., Lacau B., Cacho J., Ferreira V. (2014). Influence of viticulture practices on grape aroma precursors and their relation with wine aroma. J. Sci. Food Agric..

[3] Curiel J.A., Morales P., Gonzalez R., Tronchoni J. (2017). Different Non-*Saccharomyces* Yeast Species Stimulate Nutrient Consumption in *S. cerevisiae* Mixed Cultures. Front. Microbiol..

[4] Lambrechts M.G., Pretorius I.S. (2000). Yeast and its importance to wine aroma: A review. S. Afr. J. Enol. Vitic..

[5] Chambers P., Borneman A., Varela C., Cordente A., Bellon J., Tran T., Henschke P., Curtin C. (2015). Ongoing domestication of wine yeast: Past, present and future. Aust. J. Grape Wine Res..

[6] Boulton R.B., Singleton V.L., Bisson L.F., Kunkee E.R. (1996). Principles and Practices of Winemaking.

[7] GIL J., Mateo J., Jiménez M., Pastor A., Huerta T. (1996). Aroma Compounds in Wine as Influenced by Apiculate Yeasts. J. Food Sci..

[8] Manzanares P., Ramon D., Querol A. (1999). Screening of non-*Saccharomyces* wine yeasts for the production of β-D-xylosidase activity. Int. J. Food Microbiol..

[9] Strauss M.L.A., Jolly N.P., Lambrechts M.G., Van Rensburg P. (2001). Screening for the production of extracellular hydrolytic enzymes by non-*Saccharomyces* wine yeasts. J. Appl. Microbiol..

[10] Romano P., Fiore C., Paraggio M., Caruso M., Capece A. (2003). Function of yeast species and strains in wine flavour. Int. J. Food Microbiol..

[11] Escribano-Viana R., González-Arenzana L., Portu J., Garijo P., López-Alfaro I., López R., Santamaría P., Gutiérrez A.R. (2018). Wine aroma evolution throughout alcoholic fermentation sequentially inoculated with non- *Saccharomyces*/*Saccharomyces* yeasts. Food Res. Int..

[12] Quirós M., Rojas V., Gonzalez R., Morales P. (2014). Selection of non-*Saccharomyces* yeast strains for reducing alcohol levels in wine by sugar respiration. Int. J. Food Microbiol..

[13] Morales P., Rojas V., Quirós M., Gonzalez R. (2015). The impact of oxygen on the final alcohol content of wine fermented by a mixed starter culture. Appl. Microbiol. Biotechnol..

[14] Molina A.M., Swiegers J.H., Varela C., Pretorius I.S., Agosin E. (2007). Influence of wine fermentation temperature on the synthesis of yeast-derived volatile aroma compounds. Appl. Microbiol. Biotechnol..

[15] Scrimgeour N., Nordestgaard S., Lloyd N., Wilkes E. (2015). Exploring the effect of elevated storage temperature on wine composition. Aust. J. Grape Wine Res..

[16] Beltran G., Novo M., Guillamón J.M., Mas A., Rozès N. (2008). Effect of fermentation temperature and culture media on the yeast lipid composition and wine volatile compounds. Int. J. Food Microbiol..

[17] López-Malo M., Chiva R., Rozes N., Guillamon J.M. (2013). Phenotypic analysis of mutant and overexpressing strains of lipid metabolism genes in *Saccharomyces cerevisiae*: Implication in growth at low temperatures. Int. J. Food Microbiol..

[18] Torija M.J., Beltran G., Novo M., Poblet M., Guillamón J.M., Mas A., Rozès N. (2003). Effects of fermentation temperature and *Saccharomyces* species on the cell wall fatty acid composition and presence of volatile compounds in wine. Int. J. Food Microbiol..

[19] Massera A., Assof M., Sari S., Ciklic I., Mercado L., Jofré V., Combina M. (2021). Effect of low temperature fermentation on the yeast-derived volatile aroma composition and sensory profile in Merlot wines. LWT.

[20] Li H., Guo A., Wang H. (2008). Mechanisms of oxidative browning of wine. Food Chem..

[21] Buxaderas S., López-Tamames E., Caballero B., Trugo L., Finglas P. (2010). Wines|production of sparkling wines. Encyclopedia of Food Sciences and Nutrition.

[22] Ferreira V., Escudero A., Fernández P., Cacho J.F. (1997). Changes in the profile of volatile compounds in wines stored under oxygen and their relationship with the browning process. Z. Lebensm. Unters. Forsch..

[23] Salvadó Z., Arroyo-López F.N., Guillamón J.M., Salazar G., Querol A., Barrio E. (2011). Temperature adaptation markedly determines evolution within the genus *Saccharomyces*. Appl. Environ. Microbiol..

[24] Diaz M.R., Fell J.W. (2000). Molecular analyses of the IGS & ITS regions of rDNA of the psychrophilic yeasts in the genus *Mrakia*. Antonie Van Leeuwenhoek.

[25] Aguilera J., Randez-Gil F., Prieto J.A. (2007). Cold response in *Saccharomyces cerevisiae*: New functions for old mechanisms. FEMS Microbiol. Rev..

[26] Margesin R., Miteva V. (2011). Diversity and ecology of psychrophilic microorganisms. Res. Microbiol..

[27] Córcoles-Sáez I., Ballester-Tomas L., de la Torre-Ruiz M.A., Prieto J.A., Randez-Gil F. (2012). Low temperature highlights the functional role of the cell wall integrity pathway in the regulation of growth in *Saccharomyces cerevisiae*. Biochem. J..

[28] Turchetti B., Hall S.R.T., Connell L.B., Branda E., Buzzini P., Theelen B., Müller W.H., Boekhout T. (2011). Psychrophilic yeasts from Antarctica and European glaciers: Description of *Glaciozyma* gen. nov., *Glaciozyma martinii* sp. nov. and *Glaciozyma watsonii* sp. nov. Extremophiles.

[29] Bergauer P., Fonteyne P.-A., Nolard N., Schinner F., Margesin R. (2005). Biodegradation of phenol and phenol-related compounds by psychrophilic and cold-tolerant alpine yeasts. Chemosphere.

[30] Buzzini P., Branda E., Goretti M., Turchetti B. (2012). Psychrophilic yeasts from worldwide glacial habitats: Diversity, adaptation strategies and biotechnological potential. FEMS Microbiol. Ecol..

[31] De Garcãa V., Brizzio S., Libkind D., Buzzini P., Van Broock M. (2007). Biodiversity of cold-adapted yeasts from glacial meltwater rivers in Patagonia, Argentina. FEMS Microbiol. Ecol..

[32] Vaz A.B.M., Rosa L.H., Vieira M.L.A., De Garcia V., Brandão L.R., Teixeira L.C.R.S., Moliné M., Libkind D., Van Broock M., Rosa C.A. (2011). The diversity, extracellular enzymatic activities and photoprotective compounds of yeasts isolated in Antarctica. Braz. J. Microbiol..

[33] Carrasco M., Rozas J.M., Barahona S., Alcaíno J., Cifuentes V., Baeza M. (2012). Diversity and extracellular enzymatic activities of yeasts isolated from King George Island, the sub-Antarctic region. BMC Microbiol..

[34] Touchette D., Altshuler I., Gostinčar C., Zalar P., Raymond-Bouchard I., Zajc J., McKay C.P., Gunde-Cimerman N., Whyte L.G. (2022). Novel Antarctic yeast adapts to cold by switching energy metabolism and increasing small RNA synthesis. ISME J..

[35] Baeza M., Alcaíno J., Cifuentes V., Turchetti B., Buzzini P., Sibirny A.A. (2017). Cold-Active Enzymes from Cold-Adapted Yeasts. Biotechnology of Yeasts and Filamentous Fungi.

[36] Yusof N.A., Hashim N.H.F., Bharudin I. (2021). Cold adaptation strategies and the potential of psychrophilic enzymes from the antarctic yeast, *Glaciozyma antarctica* PI12. J. Fungi.

[37] Ballester-Tomás L., Prieto J.A., Gil J.V., Baeza M., Randez-Gil F. (2017). The Antarctic yeast *Candida sake*: Understanding cold metabolism impact on wine. Int. J. Food Microbiol..

[38] Henrici A.T. (1914). The Staining of Yeasts by Gram’s Method. J. Med. Res..

[39] Catrileo D., Acuña-Fontecilla A., Godoy L. (2020). Adaptive laboratory evolution of native *Torulaspora delbrueckii* YCPUC10 with enhanced ethanol resistance and evaluation in co-inoculated fermentation. Front. Microbiol..

[40] Ciani M., Comitini F. (2011). Non-*Saccharomyces* wine yeasts have a promising role in biotechnological approaches to winemaking. Ann. Microbiol..

[41] Dessi-Fulgheri F. (2015). Antarctic ecosystems: An extreme environment in a changing world. Ethol. Ecol. Evol..

[42] Raja H.A., Miller A.N., Pearce C.J., Oberlies N.H. (2017). Fungal Identification Using Molecular Tools: A Primer for the Natural Products Research Community. J. Nat. Prod..

[43] Güzel N., Güzel M., Savaş Bahçei K., Galanakis C.M. (2020). Nonalcoholic Beer. Trends in Non-Alcoholic Beverages.

[44] Tsuji M., Kudoh S., Hoshino T. (2016). Ethanol productivity of cryophilic basidiomycetous yeast *Mrakia* spp. correlates with ethanol tolerance. Mycoscience.

[45] Tsuji M., Tanabe Y., Vincent W.F., Uchida M. (2018). *Mrakia arctica* sp. nov., a new psychrophilic yeast isolated from an ice island in the Canadian High Arctic. Mycoscience.

[46] Linnakoski R., Jyske T., Eerikäinen R., Veteli P., Cortina-Escribano M., Magalhães F., Järvenpää E., Heikkilä L., Hutzler M., Gibson B. (2023). Brewing potential of strains of the boreal wild yeast *Mrakia gelida*. Front. Microbiol..

[47] De Francesco G., Sannino C., Sileoni V., Marconi O., Filippucci S., Tasselli G., Turchetti B. (2018). *Mrakia gelida* in brewing process: An innovative production of low alcohol beer using a psychrophilic yeast strain. Food Microbiol..

[48] Singh J., Singh R.P., Khare R. (2018). Influence of climate change on Antarctic flora. Polar Sci..

[49] Penacho V., Valero E., Gonzalez R. (2012). Transcription profiling of sparkling wine second fermentation. Int. J. Food Microbiol..

